# Patients with detectable *KIT* p.D816V in peripheral blood are at high risk for adverse systemic events during venom immunotherapy and treatment failure

**DOI:** 10.1002/clt2.70019

**Published:** 2024-12-29

**Authors:** Ajda Demšar Luzar, Jakob Otorepec, Mitja Košnik, Peter Kopač, Julij Šelb, Peter Korošec, Matija Rijavec

**Affiliations:** ^1^ University Clinic of Respiratory and Allergic Diseases Golnik Golnik Slovenia; ^2^ Biotechnical Faculty University of Ljubljana Ljubljana Slovenia; ^3^ Faculty of Medicine University of Ljubljana Ljubljana Slovenia; ^4^ Faculty of Medicine University of Maribor Maribor Slovenia; ^5^ Faculty of Pharmacy University of Ljubljana Ljubljana Slovenia

**Keywords:** clonal mast cell disease, *Hymenoptera *venom allergy, *KIT *p.D816V variant, peripheral blood leukocytes, venom immunotherapy

## Abstract

**Background:**

Recent studies have highlighted the importance of routine screening for the somatic missense *KIT* p.D816V variant in peripheral blood leukocytes (PBL), and its association with severe sting anaphylaxis. Our study aimed to evaluate the clinical relevance of *KIT* p.D816V detected in PBL on systemic adverse events (SAEs) and the efficacy of venom immunotherapy (VIT).

**Methods:**

This retrospective study included 839 patients receiving VIT. The *KIT* p.D816V variant was assayed with a highly sensitive, allele‐specific, quantitative PCR.

**Results:**

*KIT* p.D816V was detected in the PBL of 125 (15%) of 839 VIT patients. The majority (70%, 88/125) of these individuals had normal BST levels. Notably, half of the *KIT*‐positive patients receiving honeybee venom immunotherapy had SAEs during treatment (48%, 18/37; *p* = 0.0136), and the *KIT* p.D816V allele burden was higher than 0.01% in the majority of those patients (61%, 11/18). Furthermore, a significant difference was observed between *KIT*‐positive and *KIT*‐negative patients treated with VIT in the past and who experienced a recurrent reaction to a sting after treatment termination (VIT failure). *KIT*‐positive patients with VIT failure had a higher allele burden than those with successful VIT (80% vs. 40% with a *KIT* p.D816V higher than 0.01%; *p* = 0.0019). *KIT* p.D816V was a predictor of SAEs during honeybee VIT (univariate; OR = 2.43, *p* = 0.012/multivariate; OR = 2.26, *p* = 0.033) and a strong predictor of VIT failure in patients treated with wasp venom (univariate; OR = 4.1, *p* = 0.002/multivariate; OR = 3.54, *p* = 0.008).

**Conclusion:**

Our study revealed the high clinical relevance of *KIT* p.D816V detected in PBL. *KIT* p.D816V was a significant predictor of SAEs during honeybee VIT and a significant predictor of VIT failure after completing wasp VIT.

## 1 BACKGROUND

*Hymenoptera* venom allergy (HVA) is generally caused by the stings of wasps, hornets, honeybees, bumblebees, or ants. Allergic reactions to *Hymenoptera* species can present as severe manifestations and are potentially life‐threatening. The only disease‐modifying treatment available is allergen‐specific venom immunotherapy (VIT).^1^, ^2^, ^3^ Reports of systemic adverse events (SAEs) during treatment vary greatly. In a review published by Boyle et al. in 2012, the results of 11 observational studies of VIT SAE data were summarized. It was reported that the frequency of SAEs is 14% in those receiving honeybee VIT and 2.8% in those receiving wasp venom immunotherapy.^4^, ^5^ Subsequently, the frequency of SAEs during honeybee VIT was reported to be 38.7% in a study by Korošec et al. (2015),^6^ 27.9% in a study by Rosman et al. (2019)^7^ and 26.8% in a study by Kopač et al. (2021).^8^ Studies have suggested a greater percentage of SAEs during honeybee venom treatment, and honeybee venom has been suggested to be a risk factor for adverse reactions.^9^, ^10^

VIT is reportedly effective in 77%–84% of patients treated with honeybee venom and in 91%–96% of patients treated with wasp venom.^11^, ^12^ The immune tolerance established during treatment is considered to be lifelong even after discontinuation.^13^ However, in patients with known risk factors, such as severe initial sting reaction, honeybee venom allergy with high exposure to further stings, or elevated basal serum tryptase,^14^ VIT should not be discontinued. In recent decades, an association between lifetime VIT and clonal mast cell disease (CMD) has been reported.^11^, ^15^

Mastocytosis, a clonal mast cell disease, is an uncommon disease resulting from myeloid neoplasms characterized by the expansion and accumulation of clonal mast cells in one or more organ systems, including the bone marrow, skin, liver, spleen, and gastrointestinal tract. Recent studies have highlighted the importance of routine screening for the somatic missense *KIT* p.D816V variant, which is often associated with normal levels of serum tryptase and is present in the vast majority of patients with mastocytosis.^16^ This variant affects the KIT receptor of tyrosine kinase, namely, the substitution of aspartic acid with valine (D816V), which results in enhanced survival and cell growth of neoplastic mast cells, leading to several clinical manifestations.^17^ Symptoms of mastocytosis may vary from itching to profound hypotension and anaphylaxis. A recent study confirmed a strong correlation between blood and bone marrow *KIT* p.D816V allele burden, demonstrating that the presence of *KIT* p.D816V in peripheral blood, which is a minor criterion for the diagnosis of systemic mastocytosis, is highly specific for CMD.^18^

Anaphylaxis is a well‐known allergy feature of CMD, particularly venom allergy, which represents an increased risk of severe anaphylaxis.^19^, ^20^ Since the importance of routine screening for the *KIT* p.D816V variant has only recently been reported, data on the prognosis of patients treated with *Hymenoptera* venom immunotherapy with the *KIT* p.D816V variant are still being obtained. To date, the incidence of side effects during VIT in mastocytosis patients has been noted in up to 18.9%^4^, ^21^ in a 2014 study by Niedoszytko et al. and even up to 34% in CMD patients receiving wasp venom immunotherapy in a study by Jarkvist et al.^22^

It is important to note that many different factors are associated with severe sting anaphylaxis and SAEs during VIT. One of the most recently proposed independent risk factors influencing the severity of sting reactions and the course of treatment is a high basophil activation test, which reflects the basophil response to in vitro allergen stimulation, and basal serum tryptase (BST), which reflects the mast cell burden.^6^, ^8^ Increased BST could be related to an underlying hereditary α‐tryptasemia. On the other hand, BST levels below 11.4 ng/ml could be associated with the *KIT* p.D816V variant predisposing patients to CMD. The aim of our study was to evaluate the clinical relevance of the missense variant *KIT* p.D816V detected in peripheral blood leukocytes (PBLs) on SAEs and the efficacy of VIT in a larger cohort of patients. The presence of *KIT* p.D816V was determined from whole blood samples of patients receiving VIT using allele‐specific quantitative PCR. Furthermore, a statistical comparison of *KIT*‐positive and *KIT*‐negative patients was performed. Herein, we found that the *KIT* p.D816V variant is associated with a higher prevalence of SAEs during honeybee VIT and that the *KIT* p.D816V mutation is further associated with VIT failure.

## 2 METHODS

### 2.1 Study design

In this retrospective study, we included 839 patients who underwent *Hymenoptera* venom immunotherapy at the University Clinic for Respiratory Diseases and Allergy Golnik between the years 2009 and 2021 (Table 1). Patients were diagnosed and selected for VIT according to the criteria narrated by the European Academy of Allergy and Clinical Immunology.^23^ The levels of specific IgE to culprit venom and BST levels were determined. Further a diagnostic evaluation of the somatic *KIT* p.D816V variant from PBL was performed. Some patients were included in our previous studies—417 patients in a study by Lyons et al.,^24^ 33 patients in a study by Šelb et al.^16^ and nine in a study by Korošec et al.^25^ However, these studies did not evaluate the clinical relevance of *KIT* p.D816V on SAEs or the efficacy of VIT.

**TABLE 1:** Study group characteristics.

Characteristics	*n* = 839 patients
Age before VIT, median (range)	47 (17–81)
Sex, no. (%)	
Male	522 (62%)
Female	317 (38%)
Basal tryptase level (ng/ml), median (range)	4.00 (1–144)
Severity of the field sting reaction (Muller grade), no. (% of VIT)	
LLR	1 (0.1%)
I	15 (1.8%)
II	64 (7.6%)
III	301 (35.9%)
IV	458 (54.6%)
Venom immunotherapy	
Honeybee venom immunotherapy, no. (% of VIT)	290 (34%)
Wasp venom immunotherapy, no. (% of VIT)	510 (61%)
Double (honeybee and wasp) VIT, no. (% of VIT)	39 (5%)
Adverse systemic reactions during VIT	
Honeybee, no. (% of HB VIT)	100/329 (30%)
Wasp, no. (% of WVIT)	16/549 (3%)
Previous VIT^a^	
Honeybee, no. (% of HB VIT)	14/329 (4%)
Wasp, no. (% of WVIT)	20/549 (4%)
Honeybee + wasp, no. (% of VIT)^b^	29 (4%)

Abbreviations: HB, honeybee; VIT, venom immunotherapy; W, wasp.

^a^ Patients who were treated with venom immunotherapy in the past and experienced a recurrent reaction to sting after treatment termination.

^b^ Double VIT was considered.

Patients underwent honeybee or wasp ultra‐rush VIT as previously described and according to the European guidelines.^11^, ^26^, ^27^, ^28^ In the case of a systemic reaction during the build‐up phase, a slower increase in the conventional dose was adopted. The reaction grade during VIT was assigned according to the Muller grading system.^3^, ^29^ Patients who were treated with venom immunotherapy in the past with the same venom and experienced a recurrent reaction to sting after treatment termination were characterized as VIT failure. The Slovenian National Medical Ethics Committee approved the study (KME 0120/188/2017/4), and all patients provided written informed consent. Clinical data collected over the years were analysed in detail.

### 2.2 Specific IgE and total serum tryptase testing

The levels of specific IgE to culprit venom and BST levels were determined using Immulite 2000Xpi Siemens Healthcare Diagnostics (Erlangen, Germany) or ImmunoCAP immunoassay (Thermo Fisher Scientific, Waltham, Massachusetts), respectively, according to the manufacturer's instructions. Sensitization was defined as an sIgE level of 0.35 kIU/L or higher; the normal range for the total tryptase level in serum was considered to be between 1 and 11.4 ng/ml.

### 2.3 The peripheral blood *KIT* p.D816V missense variant assay

DNA was isolated using a QIAamp DNA Blood Mini Kit (Qiagen, Hilden, Germany) on a fully automated QIACube System (Qiagen). Then, DNA quantification and qualification were evaluated using a Nanodrop spectrophotometer (Thermo Fisher Scientific). The activating *KIT* c.2447A > T, p.D816V missense variant was assayed with allele‐specific quantitative PCR as described previously.^16^, ^30^, ^31^

### 2.4 Data analysis

The descriptive statistics are presented as medians and min–max ranges for measurement data and percentages for categorical data. Fisher's exact test, the chi‐square test, or the Mann‒Whitney test were used as appropriate to determine statistically significant differences using GraphPad Prism 10 software (version 10.2.1 for Windows; GraphPad Software, San Diego, CA, USA). Univariate and multivariate log regression analyses were performed using R.^32^

## 3 RESULTS

### 3.1 *KIT* p.D816V is common in *Hymenoptera* venom allergic patients with normal basal serum tryptase levels and is associated with severe *Hymenoptera* venom anaphylaxis

We evaluated the characteristics of 839 patients (522 male; median age 47 years, range 17–81 years) who were allergic to honeybee venom and/or wasp venom, received VIT and were assayed for the somatic *KIT* p.D816V variant (Table 1). In total, 290 (34%) patients were treated with honeybee VIT, 510 (61%) were treated with wasp VIT, and 39 (5%) with double VIT (honeybee and wasp venom). Higher proportions of patients with wasp VIT allergy have also been observed in previous studies.^10^, ^33^

**FIGURE 1:**
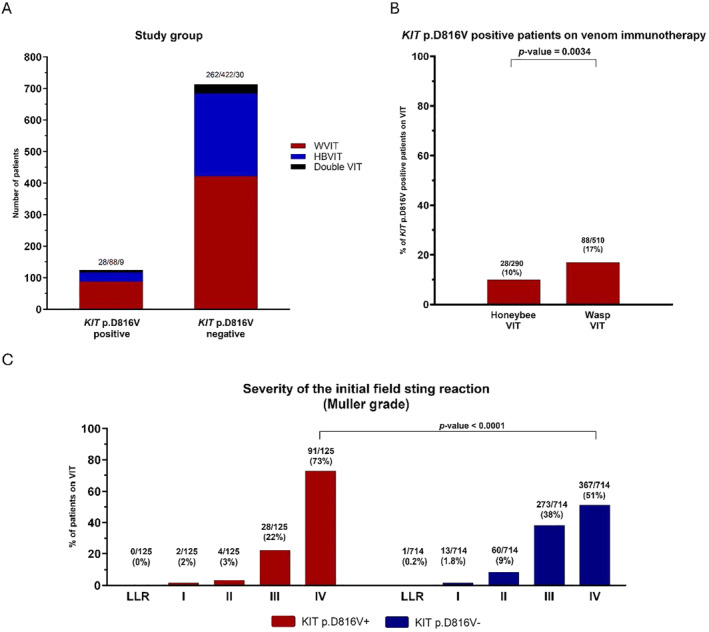
Number of *KIT*‐positive or *KIT*‐negative patients treated with honeybee, wasp, or both venom immunotherapies (A). Proportions of *KIT*‐positive patients treated with honeybee or wasp VIT (B). Muller grading of the index sting reaction of patients on VIT according to *the KIT* p.D816V variant. More severe reactions occurred in the *KIT*‐positive group of patients (C). VIT, venom immunotherapy; WVIT, wasp venom immunotherapy; HBVIT, honeybee venom immunotherapy; LLR, large local reaction.

The patients' characteristics and main results are summarized in Table 2. In total, 125 (15%) of all studied patients had detectable *KIT* p.D816V variant present in PBL (*KIT*‐positive). Interestingly, in the most recent multicentre study by Korošec et al., the prevalence of *KIT*‐positive patients treated with VIT was reported to be 21.6%,^25^ which is slightly higher than that in our cohort of patients. The median age of the *KIT*‐positive patients was higher than that of the *KIT*‐negative patients (50 vs. 46 years; *p* value = 0.0202). The significant difference observed is concordant with the suggestion that age is a risk factor for CMD. There were no significant differences between males and females. A slightly but significantly higher median BST level was observed in the group of patients with *KIT* p.D816V (6.8 ng/mL vs. 3.8 ng/mL; *p* value < 0.0001), although this value was still within the normal range. Among the 125 *KIT*‐positive patients, 88 (70%) had BST levels below 11.4 ng/mL. Our results again highlight the importance of blood screening for the *KIT* p.D816V variant since a normal BST level alone would not suggest a possible underlying CMD. The vast majority of *KIT*‐positive patients were treated with wasp VIT (88 of 125; 71%). Twenty‐eight (22%) patients were treated with honeybee venom immunotherapy, and 9 (7%) received double VIT (Figure 1A). The difference between the proportions of *KIT*‐positive patients treated with honeybee VIT and *KIT*‐positive patients treated with wasp VIT was significant (10% vs. 17%; *p* value = 0.0034) (Figure 1B, Table 2). An evaluation of the Muller grades of index stings revealed a greater proportion of grade IV reactions in comparison to other Muller grades in *KIT*‐positive patients (73% vs. 27%; *p* value < 0.0001) than in *KIT*‐negative patients (51% vs. 49%) (*p* value < 0.0001) (Figure 1C, Table 2). The observed high prevalence of the *KIT* p.D816V variant among individuals experiencing severe anaphylaxis has been described recently.^16^, ^24^, ^25^

**TABLE 2:** Clinical characteristics of the study population based on *the KIT* p.D816V variant.

Characteristics	*KIT* p.D816V positive	*KIT* p.D816V negative	*p* value
	*n* = 125	*n* = 714	
Age before VIT, median (range)	50 (20–77)	46 (17–81)	**0.0202**
Sex, no. (%)			0.0891
Male	69 (55%)	453 (63%)	
Female	56 (45%)	261 (37%)	
Basal tryptase level (ng/ml), median (range)	6.8 (1–52.2)	3.8 (1–144)	**<0.0001**
Severity of the field sting reaction (Muller grade), no. (% of *KIT*‐pos/neg)			
LLR	0	1 (0.2%)	
I	2 (2%)	13 (1.8%)	
II	4 (3%)	60 (9%)	
III	28 (22%)	273 (38%)	
IV	91 (73%)	367 (51%)	**<0.0001** **^a^**
Venom immunotherapy, no.	125	714	
Honeybee venom immunotherapy, no. (% of *KIT*‐pos/neg)	28 (22%)	262 (37%)	**0.0034**
Wasp venom immunotherapy, no. (% of *KIT*‐pos/neg)	88 (71%)	422 (59%)	
Double (honeybee and wasp) VIT, no. (% of *KIT*‐pos/neg)	9 (7%)	30 (4%)	0.1636
Adverse systemic reactions during VIT, no./HB or WVIT (% of HB or WVIT)^b^			
Honeybee	18/37 (48%)	82/292 (28%)	**0.0136**
Wasp	3/97 (3%)	13/452 (3%)	>0.999
Previous VIT, no. (% of VIT)^c^			
Honeybee	3 (8%)	11 (4%)	0.1016
Wasp	9 (9%)	11 (2%)	**0.0033**
Honeybee + Wasp^d^	10 (8%)	19 (3%)	**0.0019**

*Note*: *P* values in bold are statistically significant.

Abbreviations: HB, honeybee; VIT, venom immunotherapy; W, wasp.

^a^ Muller IV against others combined.

^b^ *p* value between honeybee and wasp *KIT* + or *KIT*‐ < 0.0001.

^c^ Patients who were treated with venom immunotherapy in the past and experienced a recurrent reaction to sting after treatment termination.

^d^ Double VIT was considered.

### 3.2 The presence of *KIT* p.D816V detected in peripheral blood leukocytes is associated with systemic adverse events during honeybee venom immunotherapy

**FIGURE 2:**
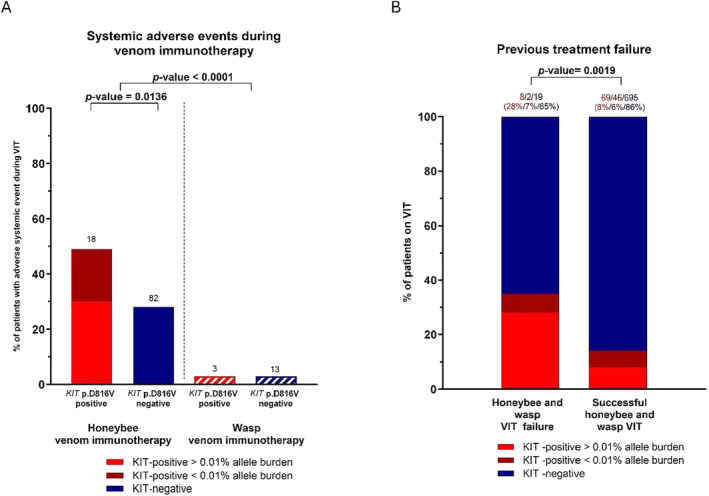
The *KIT* p.D816V variant is associated with adverse systemic events during honeybee venom immunotherapy but not during wasp venom immunotherapy (A). Compared with successful VIT, the *KIT* p.D816V variant is associated with treatment failure in roughly one‐third of honeybee and wasp VIT failure patients with higher *KIT* p.D816V variant allele burden (B). VIT, venom immunotherapy.

A clear difference in SAEs during VIT was observed between honeybee and wasp venom immunotherapy (honeybee VIT: 100/329, 30%; wasp VIT: 16/549, 3%; *p* value < 0.0001). In *KIT*‐positive honeybee‐allergic patients, SAEs during VIT occurred in 18 of 37 (48%) patients. The *KIT* p.D816V allele burden was higher than 0.01% in 11 of 18 (61%) patients (Figure 2A). In contrast, 82 of 292 (28%) *KIT*‐negative patients experienced SAEs during honeybee VIT (*p* value = 0.0136) (Table 2). Only 3 of 97 (3%) *KIT‐*positive patients and 13/452 (3%) *KIT*‐negative patients treated with wasp VIT experienced SAEs during treatment (Figure 2A). To further demonstrate the clinical relevance of the blood determined *KIT* p.D816V variant, univariate and multivariate log regression analyses showing predictors of SAEs during honeybee venom immunotherapy were performed, revealing that the *KIT* p.D816V variant was the only positive predictor of SAEs during honeybee venom treatment (univariate; OR = 2.43, *p* value = 0.012/multivariate; OR = 2.225, *p* value = 0.033) (Table 3).

**TABLE 3:** Predictors of systemic reactions during honeybee venom immunotherapy.

	Univariate log regression			Multivariate log regression		
Predictor	OR	95% CI (OR)	*p* value	OR	95% CI (OR)	*p* value
Sex	0.706	0.439–1.135	0.151	0.715	0.44–1.159	0.174
Age	1.00	0.985–1.019	0.852	0.998	0.98–1.01	0.796
BST level	1.02	0.983–1.059	0.277	1.006	0.966–1.05	0.761
Muller	1.131	0.839–1.525	0.42	1.07	0.78–1.46	0.671
*KIT* p.D816V	**2.43**	1.210–3.183	**0.012**	**2.225**	1.06–4.75	**0.033**

*Note*: *P* values in bold are statistically significant.

Abbreviations: BST, basal serum tryptase; CI, confidence interval; OR, odds ratio.

### 3.3 *KIT p.D8*16V is associated with treatment failure in one‐third of patients with VIT failure and is a strong predictor of VIT failure in patients treated with wasp venom

Evaluating previous failed treatments with immunotherapy, 3 of 37 (8%) patients with *KIT‐*positive honeybee allergies had unsuccessful treatment. Similarly, the treatment of 9 (9%) of the *KIT‐*positive patients with wasp allergies was unsuccessful. Two of these patients received double VIT (Table 2). The *KIT* p.D816V variant is associated with treatment failure in one‐third of unsuccessful honeybee and wasp VIT (VIT failure: 10 of 29, 35%; successful VIT: 115 of 695 16%; *p* value = 0.0019), with the allele burden of the *KIT* p.D816V variant being higher than 0.01% in 8 of 10 (80%) *KIT*‐positive patients (Figure 2B, Table 2). According to univariate and multivariate log regression analyses, the *KIT* p.D816V variant seems to be a strong predictor of VIT failure in wasp venom immunotherapy (univariate; OR = 4.1, *p* value = 0.002/multivariate; OR = 3.544, *p* value = 0.008) (Table 4).

**TABLE 4:** Predictors of failure of wasp venom immunotherapy.

	Univariate log regression			Multivariate log regression		
Predictor	OR	95% CI (OR)	*p* value	OR	95% CI (OR)	*p* value
Sex	1.34	0.507–3.54	0.555	1.396	0.513–3.799	0.513
Age	1.03	0.995–1.063	0.106	1.025	0.99–1.06	0.162
BST level	1.028	1.002–1.05	0.03	1.025	1.02–1.05	0.056
Muller	1.389	0.625–3.089	0.419	0.979	0.433–2.214	0.959
*KIT* p.D816V	**4.1**	1.649–10.176	**0.002**	**3.544**	1.38–9.09	**0.008**

*Note*: *P* values in bold are statistically significant.

Abbreviations: BST, basal serum tryptase; CI, confidence interval; OR, odds ratio.

## 4 DISCUSSION

Our study aimed to evaluate the clinical relevance of the *KIT* p.D816V variant detected in PBL on SAEs as well as the efficacy of VIT. To our knowledge, this is the first study to investigate these associations in a larger cohort of patients. We found that the *KIT* p.D816V variant is associated with a higher prevalence of SAEs during honeybee VIT. Furthermore, the *KIT* p.D816V variant is associated with overall VIT failure and is a strong predictor of VIT failure in patients treated with wasp venom. Importantly, the observed prevalence of *KIT*‐positive patients in our cohort of patients was 15% (125 of 839), which is similar to the most recently described prevalence observed in a large multicentre study (21.6%).^25^

An evaluation of SAEs that occurred during the course of venom immunotherapy revealed a clear difference between honeybee and wasp venom immunotherapy. Honeybee VIT is associated with more SAEs than wasp VIT. Furthermore, the *KIT* p.D816V variant detected in PBL additionally increases the probability of SAEs during honeybee VIT. To our knowledge, half of the *KIT*‐positive patients who experienced SAEs during honeybee VIT have not been previously described. Notably, the *KIT* p.D816V allele burden was higher than 0.01% in 61% of patients who experienced SAEs during honeybee VIT. With *KIT* p.D816V being a positive predictor for honeybee VIT SAEs, paying adequate attention to these patients or applying a slower and safer build‐up immunotherapy protocol should be considered.

The previously reported incidence of side effects during VIT in mastocytosis patients has been noted up to 18.9%^4^, ^21^ in a 2014 study by Niedoszytko et al. and even up to 34% in CMD patients receiving wasp venom immunotherapy, as reported in a study by Jarkvist et al.^22^ Our results suggest that a higher proportion of honeybee‐allergic patients treated with VIT might experience SAEs during VIT because the *KIT*‐positive variant predisposes patients to CMD.

Furthermore, we were interested in previous failed treatments involving the same immunotherapy. The majority of *KIT‐*positive honeybee‐allergic patients who failed previous treatment experienced SAEs during the past treatment. In contrast, a lower proportion of wasp‐allergic patients previously treated with immunotherapy with the *KIT* p.D816V variant experienced SAEs during the same past treatment. These results further suggest that *KIT* p.D816V is a positive predictor of SAEs during honeybee venom immunotherapy, even in a low number of patients, indicating that SAEs during VIT might result in unsuccessful venom immunotherapy treatment, as previously observed and proposed.^11^

Notably, the *KIT* p.D816V variant detected in PBL is associated with treatment failure in one‐third of VIT failure patients, with an even greater emphasis on wasp VIT. Moreover, a *KIT* p.D816V frequency higher than 0.01% was observed in the majority (80%) of the *KIT*‐positive VIT failure patients. This could be due to the classification of only patients who received a second immunotherapy as having VIT failure, which likely underrepresented the number of patients who experienced VIT failure.

The majority of our patients were treated with wasp VIT. This observation is expected since there is a higher proportion of patients allergic to wasp venom than to honeybee venom.^33^ Similar proportions of *Hymenoptera* venom immunotherapy were reported by Moshbech et al. in an EAACI multicentre study.^10^ Notably, most of the *KIT*‐positive patients had BST levels lower than 11.4 ng/mL. Our results further highlight the importance of *KIT* p.D816V blood screening for identifying patients with clonal CMD with normal BST levels. According to the Muller grades of index sting, a previously described^16^, ^24^, ^25^ higher proportion of severe Mueller grade IV reactions was observed in *KIT*‐positive patients than in *KIT‐*negative patients.

The main strength of our study was the large number of well‐characterized patients allergic to honeybee or wasp venom with the identified presence/absence of the *KIT* p.D816V variant. All patients received VIT following the same protocol. Therefore, we could compare *KIT*‐positive and *KIT*‐negative patients in the same clinical settings. On the other hand, in our study group, SAEs reoccurred in 4% of honeybee‐ or wasp venom‐treated patients, suggesting a higher success rate of VIT than previously described.^11^, ^12^ This could be due to the classification of only patients who received a second immunotherapy as having VIT failure, which makes it difficult to generalize the results.

Our study presents important novel findings regarding the clinical relevance of *KIT* p.D816V in *Hymenoptera* venom immunotherapy. Since *KIT* p.D816V is present in the PBL of a certain proportion of venom immunotherapy‐treated patients, it is important to acknowledge its clinical impact on SAEs and treatment success rate to ensure that it is the safest and most efficient treatment for *Hymenoptera* venom allergic patients.

## 5 CONCLUSION

This study provides the first data on associations between *KIT* p.D816V detected in peripheral blood leukocytes, SAEs during venom immunotherapy, and VIT treatment failure. Importantly, half of the *KIT* p.D816V‐positive patients who received honeybee VIT experienced SAEs during treatment. Furthermore, *KIT* p.D816V was associated with VIT failure in one‐third of the patients overall, and it was a positive predictor of wasp VIT failure. These observations emphasize the importance of *KIT* p.D816V screening before starting honeybee and wasp VIT and the need for detailed monitoring of high‐risk patients during VIT build‐up and after VIT completion.

## AUTHOR CONTRIBUTIONS

**Ajda Demšar Luzar**: Data curation; methodology; formal analysis; investigation; writing‐original draft; visualization. **Jakob Otorepec**: Data curation; investigation. **Mitja Košnik**: Methodology; resources; writing—review & editing; supervision; funding acquisition. **Peter Kopač**: Resources; writing‐review & editing. **Julij Šelb**: Resources; writing—review & editing; conceptualization; data curation; formal analysis; project administration. **Peter Korošec**: Conceptualization; methodology; writing—review & editing; visualization; supervision; funding acquisition. **Matija Rijavec**: Conceptualization; methodology; formal analysis; writing—review & editing; supervision; visualization; project administration; funding acquisition.

## CONFLICT OF INTEREST STATEMENT

The authors declare no relevant conflicts of interest.

## GRANT REFERENCES

This research was supported by the Slovenian Research and Innovation Agency (grant no. P3‐0360 and 53,537).
